# Late gene regulation by the alternative sigma factors of *Chlamydia trachomatis*

**DOI:** 10.1128/msystems.00292-25

**Published:** 2025-06-12

**Authors:** Syed M. A. Rizvi, Asha Densi, Owais R. Hakiem, Michael McClelland, Ming Tan

**Affiliations:** 1 Department of Microbiology and Molecular Genetics, University of California Irvine 8788, Irvine, California, USA; 2 Department of Medicine, University of California Irvine 8788, Irvine, California, USA; The University of Texas at Dallas, Richardson, Texas, USA

**Keywords:** transcription, gene expression, regulon, RNA polymerase, RNA-seq, ChIP-seq

## Abstract

The pathogenic bacterium *Chlamydia* reproduces via two specialized forms inside a eukaryotic host cell. The dividing form called the reticulate body (RB) must convert at late times into the infectious elementary body (EB) for spread to new host cells. Late genes are a temporal class of chlamydial genes believed to be responsible for RB-to-EB conversion, but late gene regulation is incompletely understood. In this study, we used chromatin immunoprecipitation (ChIP) to investigate two alternative sigma factors, σ^28^ and σ^54^, that alter the promoter specificity of *Chlamydia trachomatis* RNA polymerase. σ^28^ ChIP-seq identified *hctB* and *tsp* as the only promoters bound by σ^28^, and binding only occurred late, around the time of RB-to-EB conversion. Overexpression of σ^28^ confirmed that these genes are transcribed in a σ^28^-dependent manner. σ^54^ ChIP-seq showed that σ^54^ only bound *ctl0021* and *ctl0052* and only at late times. This σ^54^ regulon appears to be conserved as *in silico* analysis identified σ^54^ promoter sequences upstream of *ctl0021* and *ctl0052* homologs in all *Chlamydia* spp. The genes encoding σ^28^ and σ^54^ were only transcribed at late times, but ChIP analysis with the late regulator Euo showed that Euo only controls σ^28^ expression, and late transcription of σ^54^ is regulated in an Euo-independent manner. Thus, multiple mechanisms regulate late genes, including Euo and different forms of RNA polymerase. The dedicated use of two alternative RNA polymerases to control a small subset of late genes suggests that these genes and the independent control of their temporal expression are important for RB-to-EB conversion.

**IMPORTANCE:**

In this study, we performed chromatin immunoprecipitation-seq to identify genes transcribed by alternative forms of RNA polymerases in *Chlamydia trachomatis*. Under normal growth conditions, the sigma factors, σ^28^ and σ^54^, bound only two genes each, and binding was only detected at late times. In addition, the late regulator Euo controls the expression of σ^28^ but not σ^54^. Thus, *Chlamydia* utilizes multiple mechanisms to regulate late gene expression and uses alternative forms of RNA polymerases for specialized control of specific late genes that likely have important roles in reticulate body to elementary body conversion. This genome-wide binding approach can be applied to identify target genes of alternative sigma factors in other pathogenic bacteria.

## INTRODUCTION

*Chlamydia* is a genus of bacteria that replicates via a developmental cycle inside a eukaryotic host cell. During the 48–72 hours of intracellular infection, chlamydiae repeatedly divide and then convert into a specialized form, the elementary body (EB) (1, 2). This conversion step is critical because the replicating form, which is called a reticulate body (RB), is non-infectious, and only the EB can spread the infection to a new host cell. *Chlamydia* genes are expressed in three main temporal waves that are linked to this developmental cycle (3–6). Early genes are involved in establishing the intracellular infection, and midcycle genes are expressed during RB replication. Late genes are first transcribed at ~24 hours post-infection (hpi) when RB-to-EB conversion commences and includes genes that are critical for EB production (7–10). Transcriptomic studies have shown that temporal gene expression in *Chlamydia trachomatis* is regulated at the transcriptional level (3). The importance of transcriptional control is further demonstrated by studies showing that disruption of the transcriptional program via overexpression or depletion of transcription factors caused major defects in the developmental cycle (11, 12).

Like other bacteria, *Chlamydia* utilizes a multi-subunit enzyme called RNA polymerase to transcribe its genes (5). The core enzyme is the active component that synthesizes RNA, but the sigma (σ) subunit allows RNA polymerase to initiate the transcription of specific genes by recognizing and binding target promoters. The major form of chlamydial RNA polymerase contains σ^66^, which is the ortholog of σ^70^, the housekeeping sigma factor in *Escherichia coli* (13). *Chlamydia* also encodes two alternative sigma factors, σ^28^ and σ^54^, which substitute for σ^66^ to alter the promoter specificity of RNA polymerase. The promoter sequence recognized by *C. trachomatis* σ^28^ is similar to the *E. coli* σ^28^ promoter, and seven *C. trachomatis* promoters were transcribed by σ^28^ RNA polymerase *in vitro* (14). However, a σ^28^ overexpression and knockdown study reported only two σ^28^ target genes (15). σ^54^ belongs to a different family of sigma factors, and its promoter architecture differs from σ^66^ and σ^28^ (16). Sequences resembling bacterial σ^54^ promoters have been identified upstream of two genes in *C. trachomatis* (*ctl0021*/*ct652.1* and *ctl0052*/*ct683*) (17, 18). However, studies using σ^54^ depletion reported 67 target genes (15), and a σ^54^ activation approach identified 33 target genes (12), with little overlap in these putative σ^54^ regulated genes.

The mechanisms that regulate the delayed transcription of late genes are of special interest because they may control the timing of RB-to-EB conversion. The best-studied regulator of late gene expression is the transcription factor Euo. Euo has been shown to be a repressor of late gene promoters and has been proposed to prevent the expression of late genes until late times (5, 11). However, not all late genes are regulated by Euo, raising the possibility of additional mechanisms to control late gene expression (11). Unlike midcycle genes, promoters of late genes do not appear to be regulated by DNA supercoiling levels (19). σ^28^ and σ^54^ have been proposed to be regulators of late gene expression (12, 14, 17) because they each have a late expression pattern (3) and have putative target genes that are late genes. However, the uncertainty about their target genes makes it difficult to draw firm conclusions about the roles of the two alternative chlamydial sigma factors in late gene regulation.

In this study, we used time-course chromatin immunoprecipitation (ChIP)-seq to identify the regulons of the two alternative sigma factors in *C. trachomatis*. This approach allowed us to directly measure the binding of σ^28^ or σ^54^ to their respective target genes in the genome. Our results demonstrate that each alternative sigma factor regulates fewer genes than previously reported, but all of these target genes are late genes. Thus, *C. trachomatis* utilizes its alternative sigma factors as additional mechanisms to control late gene expression during the developmental cycle.

## MATERIALS AND METHODS

### Plasmids, strains, and antibodies

All cloning was done using NEBuilder HiFi DNA Assembly kit and the parent plasmid pBOMB4 (20). σ^28^ (*fliA*) was cloned downstream of the tet-promoter in pBOMB4 plasmid, and the resulting pBOMB4-σ^28^ plasmid was used to transform *C. trachomatis* L2 434/Bu strain for overexpression. Similarly, σ^54^ (*rpoN*) and the ATPase domain of *ctcC* (Leu_131_–Stop_387_) were cloned downstream of the conditional tet-promoter and constitutive nMen-promoter, respectively, in pBOMB4, and the resulting plasmid was used to transform *C. trachomatis* L2 434/Bu strain to overexpress a constitutively activated σ^54^ (12). A previously reported *C. trachomatis* strain (11) was used to perform σ^66^ ChIP-qPCR under Euo overexpression conditions. Available rabbit polyclonal antibodies against σ^28^ (21) and σ^54^ were used for all ChIP experiments.

### Cell culture and *C. trachomatis* transformation

Cell culture and *C. trachomatis* transformation were done as reported previously (11). HeLa cells were seeded and allowed to grow to ~90% confluency and infected with wild-type *C. trachomatis* L2 434/Bu strain at a multiplicity of infection (MOI) of ~3 by centrifugation at 750 × g for 1 hour at room temperature (RT). EBs were transformed with 5–10 µg of plasmid in 50 µL CaCl_2_ buffer.

### Total RNA extraction, cDNA synthesis, genomic DNA extraction, and RT-qPCR

Total RNA was extracted from infected HeLa cells in TRIzol reagent following the manufacturer’s instructions. The aqueous phase was collected, and total RNA was precipitated by adding an equal volume of 100% ethanol. The precipitated sample was then loaded on a silica column supplied with Qiagen’s RNeasy Mini Kit. Total RNA was eluted in 50 µL nuclease-free water. RNA was aliquoted, quantified, and stored at −80°C. cDNA was constructed using 10 µL (~1 µg) of total RNA using Bio-Rad’s iScript dDNA Clear cDNA Synthesis kit. Genomic DNA (gDNA) was collected by solubilizing infected HeLa cells in 500 µL lysis solution containing 1 mM EDTA, 10 mM Tris-HCl (pH 7.5), and 0.1% SDS, and DNA was fragmented by sonicating samples for 10 cycles of 1 second on/1 second off at 30% amplitude. For qPCR, sonicated gDNA was diluted 1:100, and 3 µL of diluted gDNA was used for each 20 µL qPCR mixture. mRNA level and genome copy number for each condition were measured by qPCR.

### Chromatin immunoprecipitation

Confluent (~90%–100%) HeLa cells in six-well cell culture plates infected with wild-type or transformed *C. trachomatis* strains at an MOI of 3, and DNA-protein interactions were fixed by incubating with 1.1% formaldehyde in 0.5 mL/well phosphate-buffered saline (PBS) for 5 minutes (σ^28^ or σ^66^) or 10 minutes (σ^54^) at RT. The reaction was quenched with 25 µL of 2.5M Glycine (RT). Fixed samples were washed (3×) with PBS at RT. For each ChIP, fixed samples from one (σ^66^) or two (σ^28^, σ^54^) six-well plates were collected in a total of 1 mL sonication buffer (1 mM EDTA, 10 mM Tris-HCl [pH 7.5], and 0.1% SDS) by scraping. Chromatin was sheared using the Covaris S220 Focused-ultrasonicator for 3 minutes for an average fragment size of ~300 bp. Dynabeads protein G magnetic beads were prepared by incubating 30 µL of a 50% slurry with polyclonal antibodies against σ^28^, σ^54^, and σ^66^ in lysis buffer for two hours at 4°C, followed by washing three times with the lysis buffer. Immunoprecipitation was done overnight by incubating sonicated lysate with the antibody-coated magnetic beads. Library was constructed using NEXTflex ChIP-seq Kit and NEXTflex barcodes (Bioo Scientific), and high-throughput sequencing was performed using Illumina Novaseq 6000 sequencing platform as reported earlier (11). Samples from all time points were sequenced to a total coverage of at least ~100-fold.

### Data analysis

High-throughput sequencing data were analyzed using Qiagen’s CLC genomics workbench (v22.0.2) software. Reads were mapped to the *C. trachomatis* L2 434/Bu genome AM884176 using high stringency settings, and ChIP peaks were called using the “transcription factor ChIP-seq” tool. Relative enrichment values were calculated by dividing the enrichment/input for the test (+Ab) by the control (−Ab) samples. Transcript level for reverse transcriptase-quantitative polymerase chain reaction (RT-qPCR) experiments was calculated using *C*_*t*_ values and PCR efficiency to normalize (22) mRNA to *C. trachomatis* gDNA levels (11).

### Prediction of σ^54^ promoters

Homer software (23) was used to perform promoter prediction and search. To examine which genes have σ^54^ promoters, we performed a BLAST analysis on coding sequences downstream of putative promoters in *C. trachomatis* L2/434/Bu genome to identify their *C. trachomatis* orthologs.

## RESULTS

### Identification of the σ^28^ regulon

**Fig 1:**
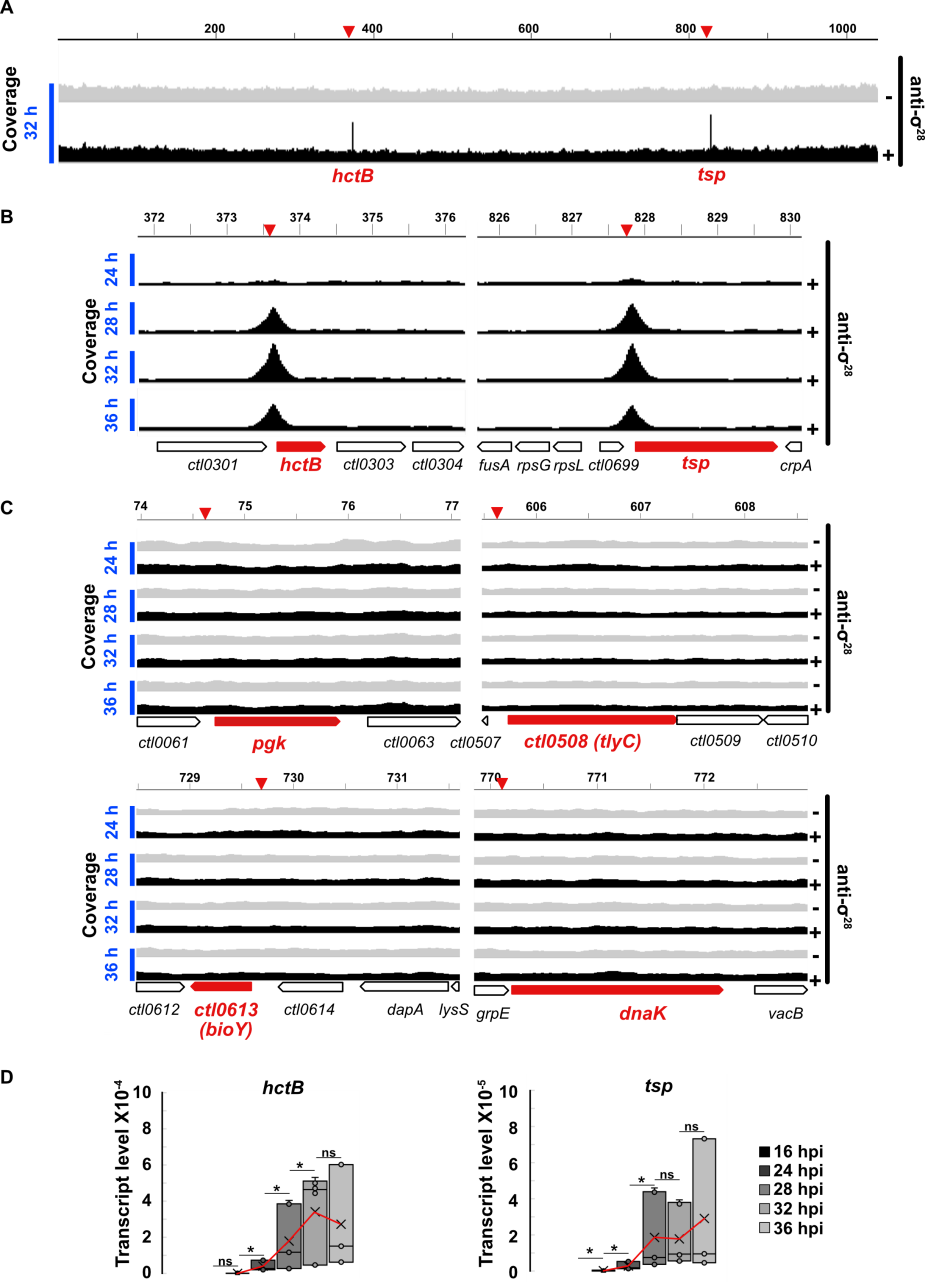
(**A**) Coverage of mapped reads showing ChIP-peaks in *hctB* and *tsp* promoter regions at 32 hpi. Light gray shows the coverage of no-antibody negative control, and black shows coverage for immunoprecipitated samples. (**B**) Temporal change in the binding of σ^28^ at the promoters of *hctB* and *tsp*. (**C**) The absence of σ^28^ ChIP-peaks in the promoter regions of previously identified σ^28^ transcribed genes. Genes are shown in red. Inverted wedges (red) on top of each panel show the expected location of the ChIP-peak in the promoter region. (**D**) Temporal expression of *hctB* (left) and *tsp* (right) measured as a ratio of mRNA to gDNA. Red line traces the mean transcript level. *P < 0.05, ns: not significant, *t*-test, *n* = 3. Genomic loci are shown in kb.

**Fig 2:**
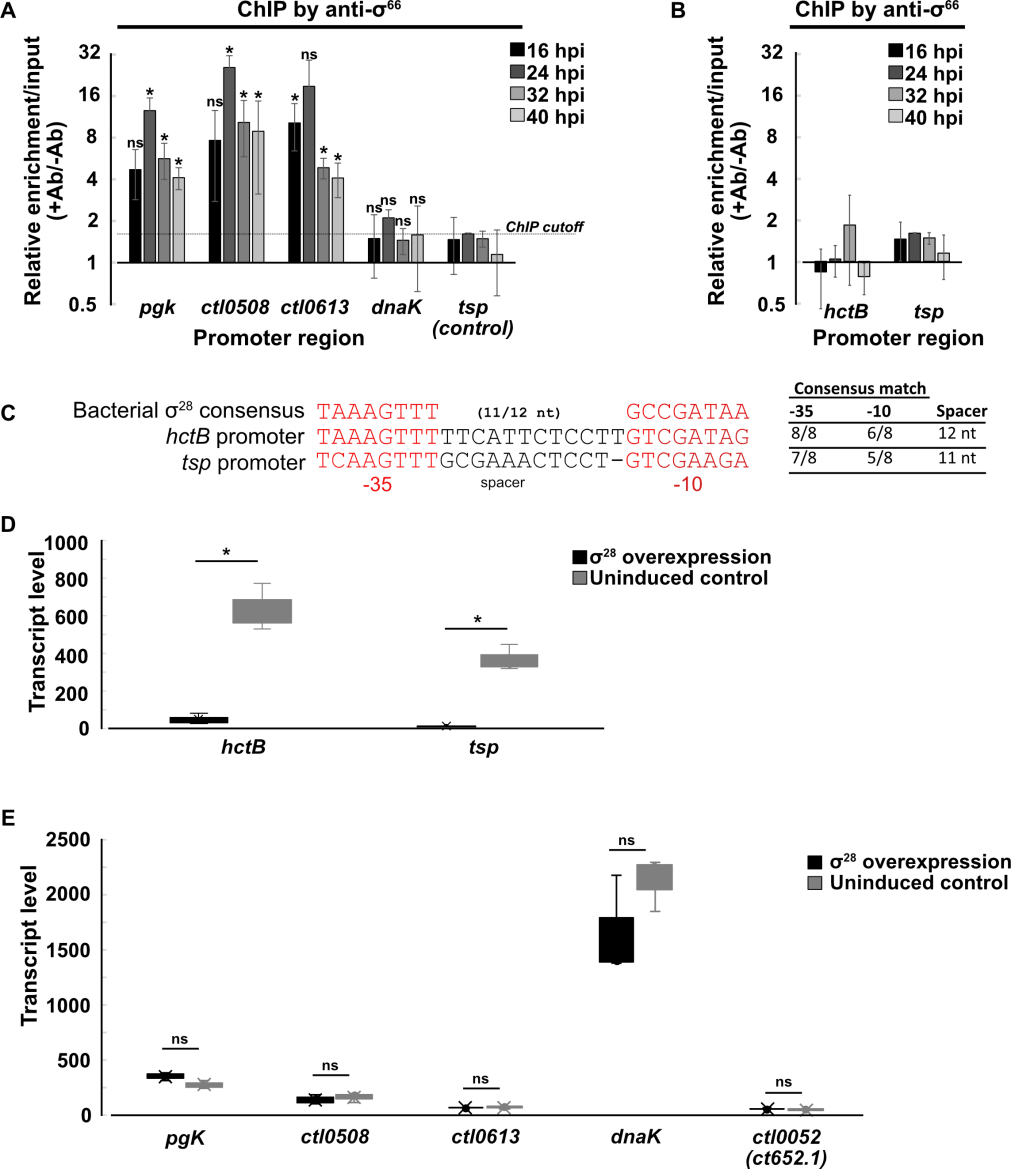
(**A**) ChIP-qPCR showing binding of σ^66^ at the promoters of *pgk*, *ctl0508*, *ctl0613*, and *dnaK*. Relative enrichment is measured as the ratio of enrichment when anti-σ^66^ antibodies (+Ab) are used vs mock enrichment with beads without antibody (−Ab). The specificity of σ^66^ ChIP is shown by the absence of enrichment of the promoter region of σ^28^ transcribed gene *tsp*. *P < 0.05, *t*-test, *n* = 2. (**B**) ChIP-qPCR showing binding of σ^66^ at the promoters of *tsp* and *hctB*. (**C**) Comparison of *hctB* and *tsp* promoters with the consensus bacterial σ^28^ promoter. (**D**) Overexpression of σ^28^ resulted in increased expression of *hctB* and *tsp* compared to wild type (un-induced control). *P < 0.05, *t*-test, *n* = 3. (**E**) No increase was observed in the expression of *pgk*, *ctl0508*, *ctl0613*, or *ctl0052*.

To identify genes that are transcribed by σ^28^ RNA polymerase, we performed a σ^28^ ChIP-seq analysis on HeLa cells infected with *C. trachomatis* serovar L2. This approach detects σ^28^ binding sites in the *C. trachomatis* genome at the time of analysis in the intracellular infection. Using polyclonal antibodies against σ^28^, we immunoprecipitated DNA fragments bound by σ^28^, which were then identified by deep sequencing. σ^28^ ChIP-seq analysis at 32 hpi revealed that σ^28^ only bound two sites in the *C. trachomatis* genome. These ChIP-peaks were located at *hctB* and *tsp* (Fig. 1A), which are genes that have been reported to be σ^28^ targets (14, 15). We then performed a time course σ^28^ ChIP-seq from 24 to 36 hpi, which revealed that σ^28^ bound *hctB* and *tsp* at 28 hpi and later time points, with maximum binding at 32 hpi, but no σ^28^ binding was detected at 24 hpi (Fig. 1B; Fig. S1A). The σ^28^ ChIP-peaks for *hctB* and *tsp* were both centered upstream of the gene, around the −10 element of their experimentally defined σ^28^ promoters (14) (Fig. 2C).

We did not detect any other σ^28^ ChIP peaks in our σ^28^ ChIP-seq analysis at any time point (Fig. S1A). In particular, we did not detect σ^28^ ChIP-peaks for *pgk*, *ctl0508 (tlyC*), *ctl0613 (bioY*), and *dnaK*, which are *C. trachomatis* genes transcribed by σ^28^ RNA polymerase *in vitro* (14) (Fig. 1C). In contrast, a parallel σ^66^ ChIP-seq analysis showed that promoter regions upstream of *pgk*, *ctl0508*, and *ctl0613* were each enriched for σ^66^ binding (Fig. 2A). The promoter region upstream of *dnaK* was not enriched for σ^66^ binding, suggesting that this gene does not have its own internal promoter within the *hrcA*-*grpE*-*dnaK* operon. These findings provide evidence that these putative σ^28^ targets are instead transcribed by σ^66^ RNA polymerase and not σ^28^ RNA polymerase under the growth conditions tested. In contrast, the *tsp* or *hctB* promoter regions (Fig. 2B) were not enriched for σ^66^ binding (Fig. 2B).

We performed RT-qPCR on *C. trachomatis*-infected cells to check if the late σ^28^ binding pattern at *hctB* and *tsp* correlated with the temporal expression patterns of these genes. We measured the greatest increase in *hctB* and *tsp* transcript levels between 24 and 28 hpi (Fig. 1D and E), concordant with the onset of σ^28^ binding between 24 hpi and 28 hpi in our ChIP-seq analysis (Fig. 1B top two panels). For additional evidence that *hctB* and *tsp* are regulated by σ^28^, we generated a *C. trachomatis* σ^28^ overexpression strain in which exogenous expression of σ^28^ is controlled by the addition of anhydrotetracycline to the tissue culture medium (11). Overexpression of σ^28^ during midcycle increased transcript levels of *hctB* and *tsp* (Fig. 2D) but not the four putative σ^28^ targets that σ^28^ did not bind in our ChIP assay (Fig. 2E). Collectively, these results provide direct evidence that σ^28^ binds to the promoters of *hctB* and *tsp* during the *C. trachomatis* developmental cycle.

### The σ^28^ gene is transcribed by σ^66^ RNA polymerase and regulated by Euo

**Fig 3:**
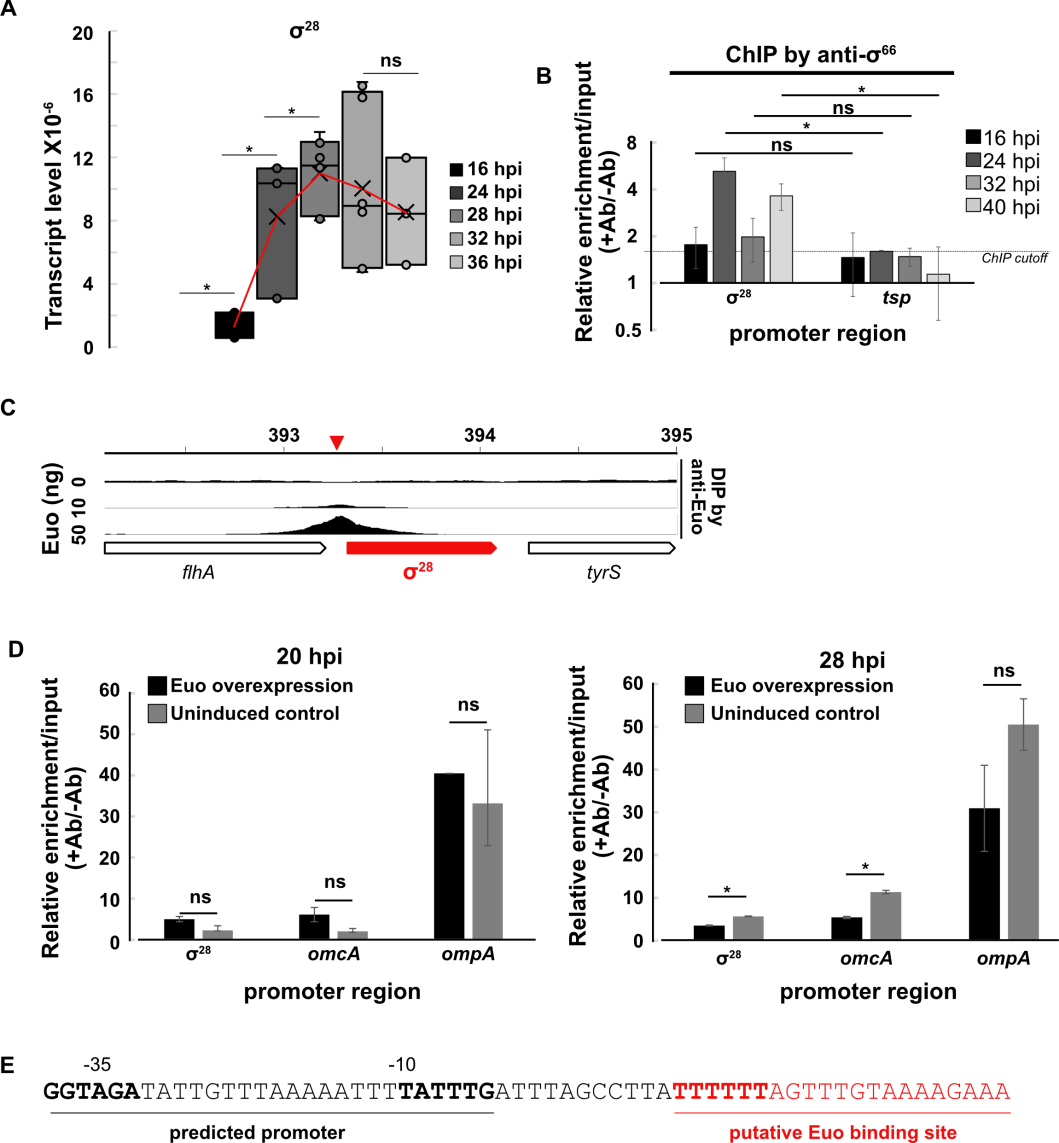
(**A**) Temporal expression of σ^28^ was measured as the number of transcripts normalized to bacterial gDNA. Red line traces the mean transcript level. (**B**) ChIP-qPCR showing increase in σ^66^ binding at the promoter of σ^28^ after 16 hpi. (**C**) DIP showing binding of Euo at σ^28^ promoter using 10 and 50 ng purified recombinant Euo. *P < 0.05, *t*-test, *n* = 3. (**D**) ChIP-qPCR shows reduced binding of σ^66^ in the promoter region of σ^28^ and om*cA* but not *ompA* due to Euo overexpression at 28 hpi but not at 20 hpi. (**E**) Predicted σ^66^ promoter sequence upstream of the coding region of the σ^28^ gene and putative Euo-binding site downstream of the promoter. *P < 0.05, *t*-test, *n* = 2.

The late patterns of σ^28^ binding and σ^28^-regulated gene expression prompted us to investigate how σ^28^-dependent transcription is temporally regulated. Using RT-qPCR, we detected transcription of the σ^28^ gene (*fliA*), from 24 hpi onward but not at 16 hpi (Fig. 3A). In addition, σ^66^ ChIP-qPCR only detected enrichment of σ^66^ at the σ^28^ promoter region at 24 hpi and later times (Fig. 3B), indicating transcription by σ^66^ RNA polymerase at late times. Since Euo bound the σ^28^ promoter region in our previous Euo DNA immunoprecipitation (DIP) analysis (Fig. 3C) (11), we investigated if this late transcription of σ^28^ is controlled by Euo. Using a *C. trachomatis* Euo overexpression strain (11), we performed a σ^66^ ChIP-qPCR analysis and found that exogenous Euo expression significantly reduced σ^66^ binding at the σ^28^ promoter at 28 hpi (Fig. 3D), consistent with Euo-mediated repression. Together, these results demonstrate that the σ^28^ gene is transcribed by σ^66^ RNA polymerase, but transcription only occurs at late times because of temporal regulation by Euo. Intriguingly, Euo overexpression only reduced σ^66^ binding to σ^28^ and another Euo-regulated gene *omcA*, at a late time (28 hpi) and not during midcycle (20 hpi; Fig. 3D). This observation suggests that endogenous Euo levels are sufficient to repress its target genes in midcycle but are limiting at late times, which allows σ^66^ RNA polymerase to transcribe σ^28^ and other Euo-regulated late genes. A putative σ^66^ promoter and a putative Euo operator were identified upstream of the σ^28^ coding region (Fig. 3E).

### Identification of the σ^54^ regulon

**Fig 4:**
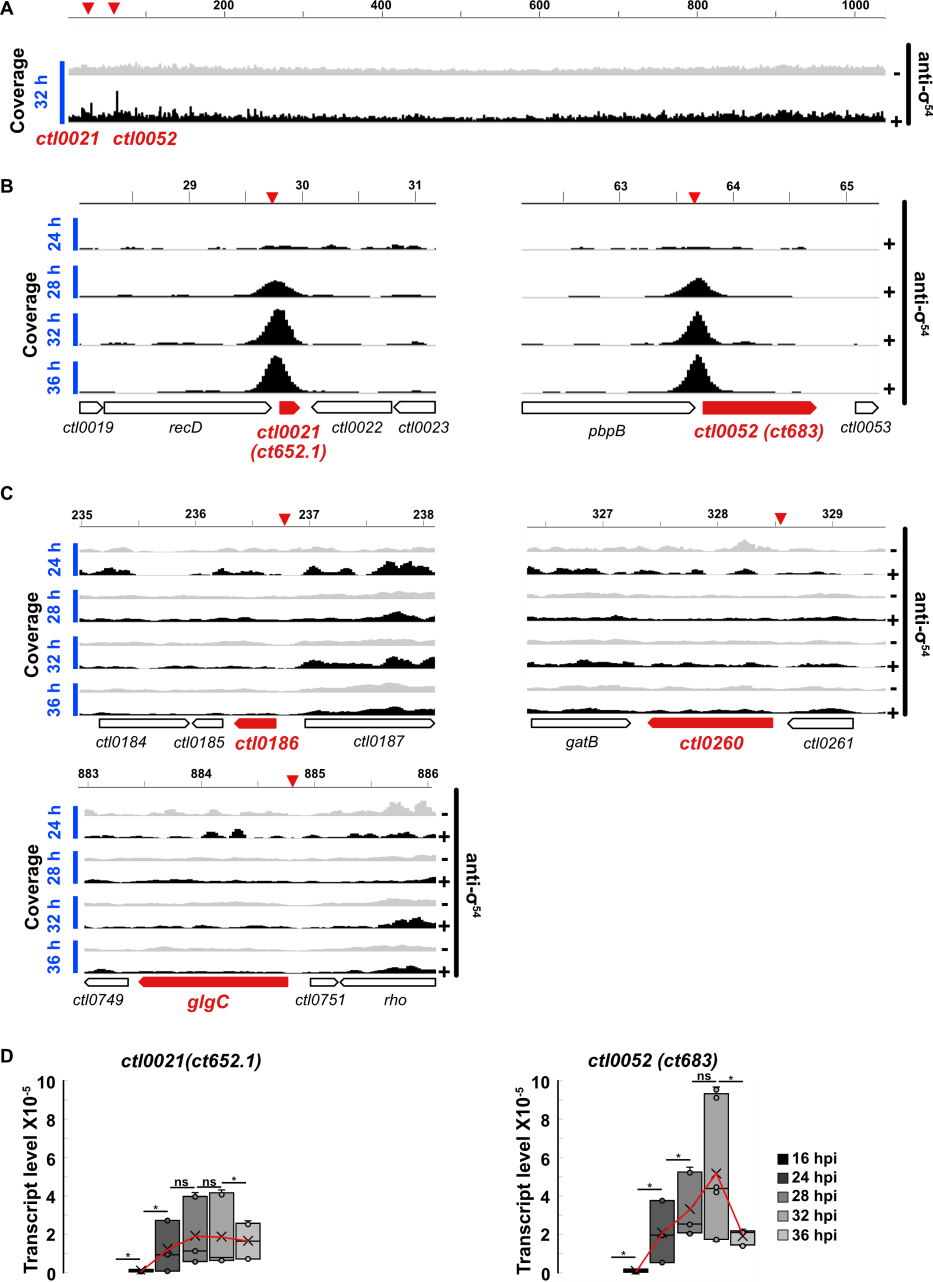
(**A**) Coverage of mapped reads showing ChIP-peaks in *ctl0021* and *ctl0052* promoter regions at 24, 28, 32, and 36 hpi. Light gray shows the coverage of no-antibody negative control, and black shows coverage for immunoprecipitated samples. (**B**) Temporal change in the binding of σ^54^ in the promoters of *ctl0021* and *ctl0052*. (**C**) The absence of σ^54^ ChIP-peaks in the promoter region of *ctl0186*, *ctl0260*, and *glgC*. These genes previously identified as σ^54^ transcribed genes are shown in red. Inverted red wedges on top of each panel show the expected location of ChIP-peaks in the promoter region. (**D**) Temporal expression of *ctl0021* (left) and *ctl0052* (right) was measured as a ratio of mRNA to gDNA. Red line traces the mean transcript level. *P < 0.05, *t*-test, *n* = 3.

Taking a similar approach, we performed a ChIP-seq time course to identify genes transcribed by σ^54^ RNA polymerase in *C. trachomatis* serovar L2-infected HeLa cells. ChIP-seq with anti-σ^54^ antibodies at 32 hpi revealed that σ^54^ only bound two sites in the *C. trachomatis* genome (Fig. 4A). These sites were located at *ctl0021* and *ctl0052 (ct652.1* and *ct683*, respectively, in *C. trachomatis* serovar D), which are the two best characterized σ^54^ promoters in *C. trachomatis* (17). σ^54^ binding to *ctl0021* and *ctl0052* was detected at 28 hpi and later, with maximum binding at 32 hpi and 36 hpi, but no binding was observed at 24 hpi (Fig. 4B; Fig. S1B). We did not detect σ^54^ binding to other genes at any time point from 24 to 36 hpi (Fig. S1B), including the ~100 genes that have been reported as σ^54^-regulated genes in *C. trachomatis* (12, 15). Our initial σ^54^ ChIP-seq assay was performed with sequencing coverage of ~50-fold, but we did not detect any additional σ^54^ ChIP peaks when we increased the depth of sequencing to achieve a coverage of more than 150-fold. The two σ^54^ ChIP-peaks were centered around ~50 bp upstream of the *ctl0021* and *ctl0052* coding regions, respectively (Fig. 4B). Consistent with this late σ^54^ binding pattern, *ctl0021* and *ctl0052* both had a late transcription pattern, as measured by RT-qPCR (Fig. 4D). The expression of both *ctl0021* and *ctl0052* increased significantly between 16 and 24 hpi, plateaued between 24 and 32 hpi, then decreased after 32 hpi.

### Analysis of reported σ^54^ target genes that were not detected in our σ^54^ ChIP assay

**Fig 5:**
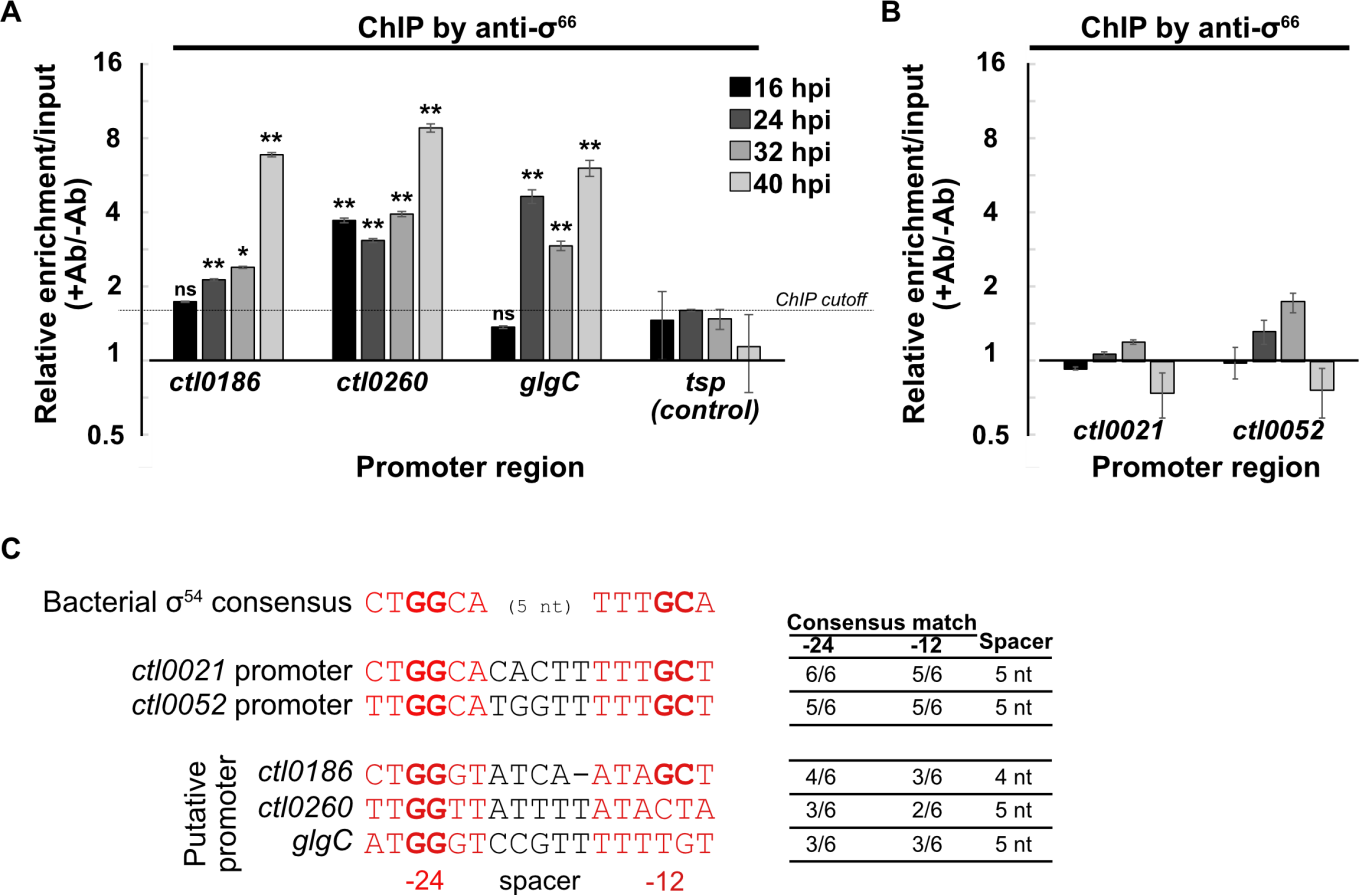
(**A**) ChIP-qPCR showing binding of σ^66^ at the promoters of *ctl0186*, *ctl0260*, and *glgC*. Promoter region of σ^28^ transcribed gene *tsp* is used as a negative control. Relative enrichment is measured as the ratio of enrichment using anti-σ^66^ antibodies (+Ab) to mock enrichment with beads without antibody (−Ab). The promoter of σ^28^ transcribed gene *tsp* was not bound by σ^66^. *P < 0.05, **P < 0.005, *t*-test, *n* = 2. (**B**) ChIP-qPCR showing no binding of σ^66^ at the promoters of *ctl0021* and *ctl0052*. (**C**) Comparison of *ctl0021* and *ctl0052* promoters with the consensus bacterial σ^54^ promoter.

To investigate the large discrepancy between the number of σ^54^ targets identified in our ChIP-seq analysis and previous RNA-seq studies, we selected three genes (*ctl0186*, *ctl0260*, and *glgC*) for further evaluation. These putative σ^54^ target genes were selected because they are upregulated at a late time and have the best available evidence from the Soules et al. (12) and Hatch and Ouellette reports (15), based on identification in both studies, higher levels of differential expression in response to σ^54^ depletion, and potential σ^54^ promoters identified bioinformatically (15). However, in our ChIP-seq time course, none of these genes had significant σ^54^ binding compared to a no-antibody negative control at any time point (Fig. 4C). Sequence analysis showed that the putative σ^54^ promoters of these genes each had significant differences at critical positions (GG in the −24 promoter element and GC in the −12 element) or deviation from the optimal 5 bp spacer length compared to the consensus bacterial σ^54^ promoter sequence (Fig. 5C) (24). In contrast, the predicted σ^54^ promoters of our σ^54^ targets, *ctl0021* and *ctl0052*, showed a 100% match to these critical bases and spacer length compared to the consensus σ^54^ sequence (17). For further analysis, we performed a parallel ChIP-qPCR using anti-σ^66^ antibodies to determine if these genes are transcribed by σ^66^ RNA polymerase. We detected σ^66^ binding, as measured by σ^66^ enrichment, to *ctl0186*, *ctl0260*, and *glgC* (Fig. 5A)*,* but not our σ^54^ targets, *ctl0021* and *ctl0052* (Fig. 5B). Collectively, these findings provide evidence that *ctl0021* and *ctl0052* are transcribed by σ^54^ RNA polymerase, but *ctl0186*, *ctl0260*, and *glgC* are transcribed by σ^66^ RNA polymerase and not σ^54^ RNA polymerase.

### The σ^54^ gene is transcribed by σ^66^ RNA polymerase but not regulated by Euo

**Fig 6:**
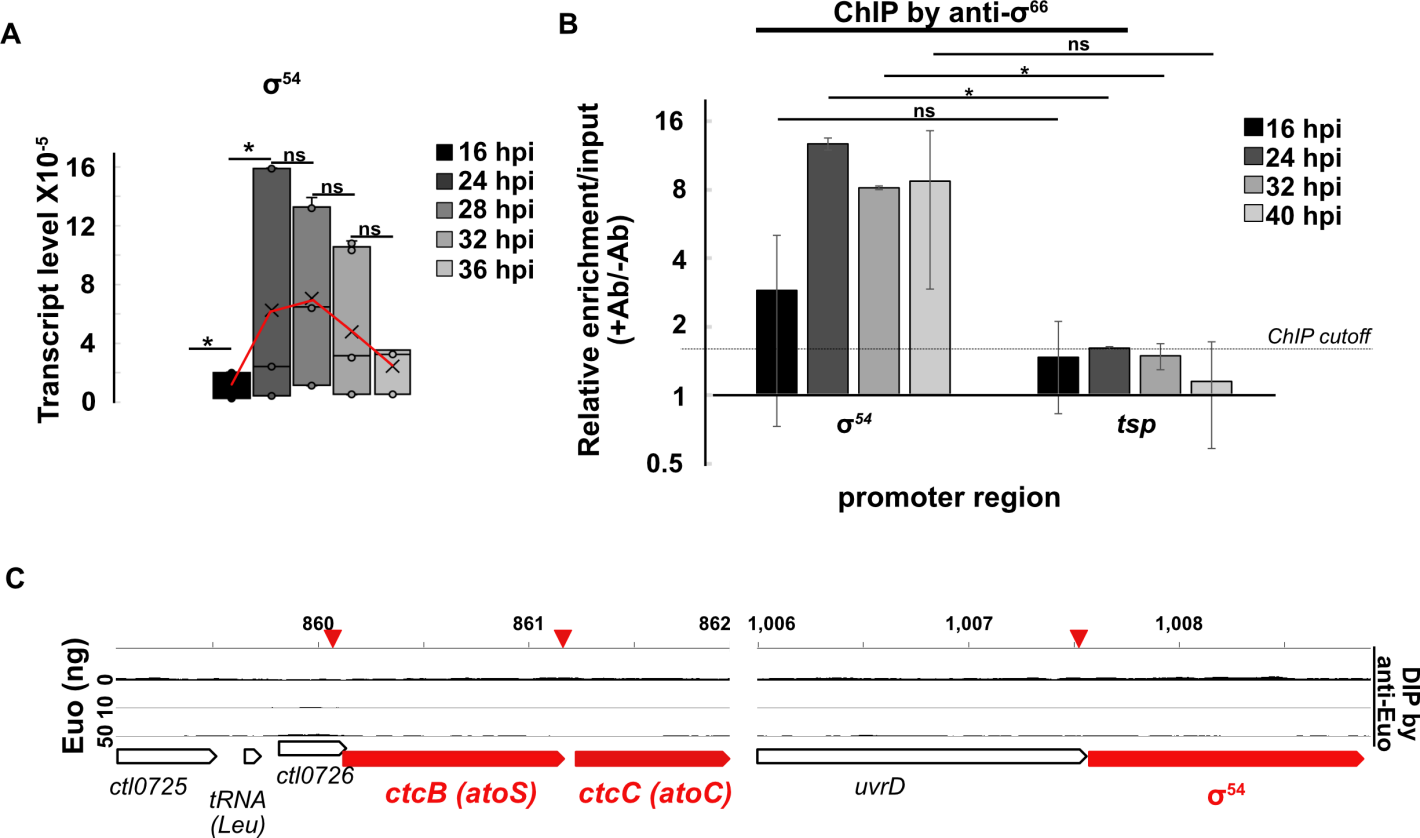
(**A**) Temporal expression of σ^54^ measured as the number of transcripts normalized to bacterial gDNA. Red line traces the mean transcript level. *P < 0.05, *t*-test, *n* = 3. (**B**) ChIP-qPCR showing increase in σ^66^ binding at the promoter of the σ^54^ gene after 16 hpi. *P < 0.05, *t*-test, *n* = 2. (**C**) DIP shows no binding of Euo in the putative promoter region of *ctcB*, *ctcC*, or σ^54^. Inverted red wedges on top of the panels show the expected location of Euo DIP-peaks if Euo binds these genes.

We performed RT-qPCR to measure transcripts from the σ^54^ gene (*rpoN*) and detected maximal expression at late times (24 and 28 hpi; Fig. 6A). ChIP-qPCR with anti-σ^66^ antibodies showed enrichment for σ^66^ in the promoter region upstream of the σ^54^ gene (Fig. 6B). In contrast, neither σ^28^ nor σ^54^ bound this promoter region in ChIP-seq analyses with anti-σ^28^ and anti-σ^54^ antibodies (Fig. S1). These results indicate that the σ^54^ gene is transcribed at late times by σ^66^ RNA polymerase. We then investigated if σ^54^ or its predicted regulators are controlled by the late regulator Euo. The examination of our published Euo DIP peak analysis showed no Euo DIP peaks in the promoter regions of the σ^54^ gene, or *ctcB* (*atoS*) or *ctcC* (*atoC*; Fig. 6C), which encode putative two-component regulators of *C. trachomatis* σ^54^. Moreover, we did not detect differential expression of σ^54^, *ctcB*, or *ctcC* in a previous report where we performed differential RNA-seq in response to Euo overexpression (11). Thus, Euo does not appear to be responsible for the late expression of σ^54^ or the CtcB/CtcC activation system. Overall, these results show that the σ^54^ gene is transcribed by σ^66^ RNA polymerase, but the late expression or activation of σ^54^ is regulated by an additional mechanism other than Euo.

### *In silico* prediction of σ^54^ regulated genes in *Chlamydia* and *Chlamydia*-related bacteria

**Fig 7:**
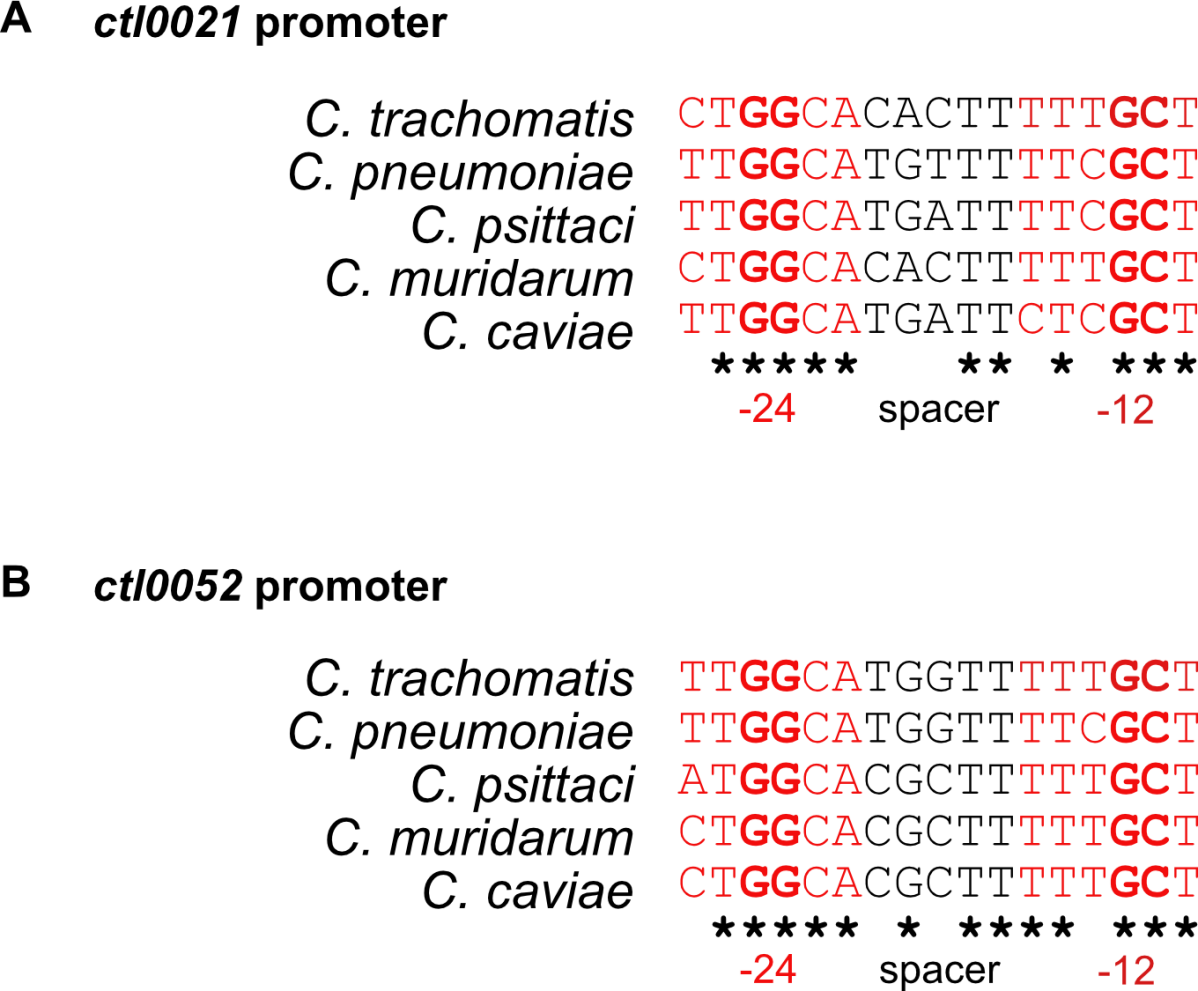
σ^54^ promoter-like sequences identified upstream of *ctl0021* (**A**) and *ctl0052* (**B**) homologs in *C. pneumoniae* (*CWL029*), *Chlamydia psittaci* (*Cps_WRSTE30*), *Chlamydia muridarum* (*Cmu_Nigg3*), and *Chlamydia caviae* (*Cca_GPIC*) are conserved with all the critical bases (bold) matching in the −12 and −24 (red) elements of the σ^54^ promoters *C. trachomatis* (*Ctr_434/Bu*) and ideal spacer lengths (5 bp) maintained between the −12 and −24 elements.

To investigate if σ^54^ has a conserved role in regulating chlamydial gene expression, we performed an *in silico* analysis to identify σ^54^-regulated genes in other *Chlamydia* spp. and *Chlamydia*-related bacteria. We based this analysis on *ctl0021* rather than *ctl0052* because Ctl0021 is conserved in all sequenced *Chlamydia* and *Chlamydia*-related bacteria, with only one exception (Table S1). We aligned the non-coding regions immediately upstream of each *ctl0021* gene and identified a putative σ^54^ promoter in every case (Table S1). We then used these promoter sequences to define a consensus sequence (TGGCACAATTTTCGCT), position weight matrix, and a selection cutoff, enabling us to predict σ^54^ promoters using the promoter prediction tool Homer (23). When we applied this prediction analysis to the *C. trachomatis* L2/434/Bu genome, we successfully predicted the σ^54^ promoter of *ctl0052*, along with a few hits that are likely to be false positives because they were within coding regions. We then extended this promoter prediction to other *Chlamydia* spp. and *Chlamydia*-related bacteria, limiting our analysis to promoter-like sequences in non-coding regions. Almost all the predicted promoters were upstream of *ctl0021* and *ctl0052* orthologs (Fig. 7; Table S1). A notable exception was *Chlamydia pneumoniae*, which had σ^54^ promoter-like sequence upstream of *ftsK* in addition to *ctl0021* and *ctl0052*. Two *Chlamydia*-related bacteria, ca. *Protochlamydia amaebophila* and ca. *Protochalmydia naegleriophila*, appear to lack *ctl0052* but had σ^54^ promoter-like sequence upstream of *ctl0021* and additional genes not found in *C. trachomatis*. This *in silico* analysis provides evidence of a transcriptional unit composed of the alternative sigma factor σ^54^ and its target genes, *ctl0021* and *ctl0052*, conserved in most *Chlamydia* spp. and *Chlamydia*-related bacteria.

## DISCUSSION

In this study, we used time-course ChIP-seq and additional data to identify genes bound by alternative sigma factors during the *C. trachomatis* developmental cycle. We found that σ^28^ and σ^54^ each bound two genes and only late in the intracellular infection. The small number of σ^28^ target genes is consistent with a published report (15), but we found evidence for far fewer σ^54^ target genes than previous studies, as will be discussed further (12, 15). The temporal pattern of σ^28^ and σ^54^ binding can be explained by the late expression of the genes encoding these alternative sigma factors. However, only the σ^28^ gene was regulated by the late regulator Euo, and an unknown Euo-independent mechanism controls the temporal expression of the σ^54^ gene. These findings indicate that there are multiple mechanisms to regulate late gene expression in *Chlamydia*.

ChIP-seq provides several advantages for identifying target genes regulated by a specific form of chlamydial RNA polymerase (25–27). Because it is a direct binding assay, ChIP provides stronger evidence than differential expression of a gene, which could be due to direct regulation by the sigma factor or an indirect effect via another factor. The cross-linking step in ChIP preserves the physical interaction between the sigma factor and each target gene, which allows binding to be measured within the bacterium, even inside a host cell. Binding can thus be measured during the normal developmental cycle, unlike approaches that induce differential expression but may introduce other changes to the intracellular infection. By performing the cross-linking step at different times in the developmental cycle, we were able to show that σ^28^ and σ^54^ only bound at late times. In addition, this ChIP analysis could be performed under different growth conditions, such as chlamydial persistence or hypoxia, to measure their effects on transcription. A further advantage is in promoter identification because the σ subunit binds the promoter of the gene—where RNA polymerase initiates transcription—but not the coding region since σ is released with promoter clearance (28).

We performed additional studies to bolster our ChIP-seq target identification. For σ^28^, we used a *C. trachomatis* σ^28^ overexpression strain to show that transcription of *hctB* and *tsp* was σ^28^-dependent (Fig. 2D). Published *in vitro* and differential RNA-seq studies have also identified *hctB* and *tsp* as σ^28^-transcribed genes (14, 15, 21). However, four other reported genes with σ^28^-transcribed promoters do not appear to be *bona fide* targets because they lacked σ^28^ binding (Fig. 1C) and σ^28^-dependent transcription in chlamydiae (Fig. 2E) and were not detected in a σ^28^ overexpression/knockdown transcriptomic study (15). This discrepancy demonstrates the limitations of identifying target genes based on transcription by σ^28^ RNA polymerase *in vitro* or in a heterologous system (14, 29). An additional drawback of *in vitro* or reporter assays is that they are usually analyzed with a single promoter in isolation, without competition from other promoters in the genome. These four genes are likely transcribed by σ^66^ RNA polymerase as they were bound by σ^66^ (Fig. 2A).

There are good data to support the two σ^54^ target genes that we identified. *ctl0021* and *ctl0052* each have promoter sequences that are well conserved with the consensus bacterial σ^54^ promoter sequence (Fig. 5C). These promoter sequences are in the correct location upstream of *in vivo* transcription start sites mapped in *C. trachomatis* by Timms and colleagues (17), whereas transcription start sites have not been verified for other putative σ^54^ promoters. We were unable to generate a *C. trachomatis* σ^54^ overexpression strain, which requires expression of σ^54^ and activation by the CtcB/CtcC two-component system (30). However, we showed a temporal correlation between σ^54^ binding (Fig. 4B) and the late transcription of *ctl0021* and *ctl0052* in *C. trachomatis*-infected cells (Fig. 4D). In addition, we used a stringent *in silico* analysis to identify well-conserved σ^54^ promoter sequences upstream of *ctl0021* and *ctl0052* homologs in all *Chlamydia* spp. and almost all *Chlamydia*-related bacteria (Fig. 7; Table S1). Collectively, these data provide evidence that *ctl0021* and *ctl0052* are regulated by σ^54^ as a transcriptional unit conserved in the order Chlamydiales.

Approximately 100 σ^54^ target genes have been proposed for *C. trachomatis* but were not bound by σ^54^ in our ChIP-seq study. These genes were identified on the basis of differential transcription in response to exogenous expression of the putative σ^54^ activator, CtcC (12), or to knockdown of σ^54^ (15). However, transcriptional changes were detected by RNA-seq, which does not indicate if the transcripts were produced by σ^54^ RNA polymerase or other chlamydial RNA polymerases. Importantly, differential expression may not have been specific for σ^54^ because σ^54^ activation and knockdown both disrupted the progression of the developmental cycle (12, 15). The likelihood of non-specific differential expression is further suggested by the inability of the σ^54^ knockdown RNA-seq study to identify either *ctl0021* or *ctl0052* as targets (15) and for the σ^54^ activation study to only identify *ctl0052* and not *ctl0021* (12). In addition, we postulate that σ^54^ knockdown may not have altered σ^54^-dependent transcription because it was measured at 24 hpi—a time in the developmental cycle before σ^54^ binding is detected (Fig. 4B; Fig. S1B).

We performed additional analyses to investigate this discordance in the identification of σ^54^ target genes. We increased the depth of sequencing for our ChIP-seq analysis by 150-fold but still did not detect σ^54^ binding to genes other than *ctl0021* or *ctl0052*. Furthermore, our ChIP-PCR analysis of *ctl0186*, *ctl0260*, and *glgC*, which are putative σ^54^ target genes with the best-supporting evidence, showed that they are transcribed by σ^66^ RNA polymerase rather than σ^54^ RNA polymerase. We did not analyze all the other proposed σ^54^ target genes, but our ChIP-seq study casts considerable doubt on them, and additional experimental data are required to show that they are *bona fide* σ^54^ targets.

σ^54^ RNA polymerase could possibly transcribe additional genes besides *ctl0021* and *ctl0052* under conditions not included in our analysis, but differential expression of these genes would require a target-specific σ^54^ regulator, which has not yet been identified in *Chlamydia*. In contrast, CtcC, the putative activator of *Chlamydia* σ^54^, is likely to activate σ^54^ RNA polymerase in a general manner because it lacks a DNA-binding domain for selective binding to specific target genes (31).

**Fig 8:**
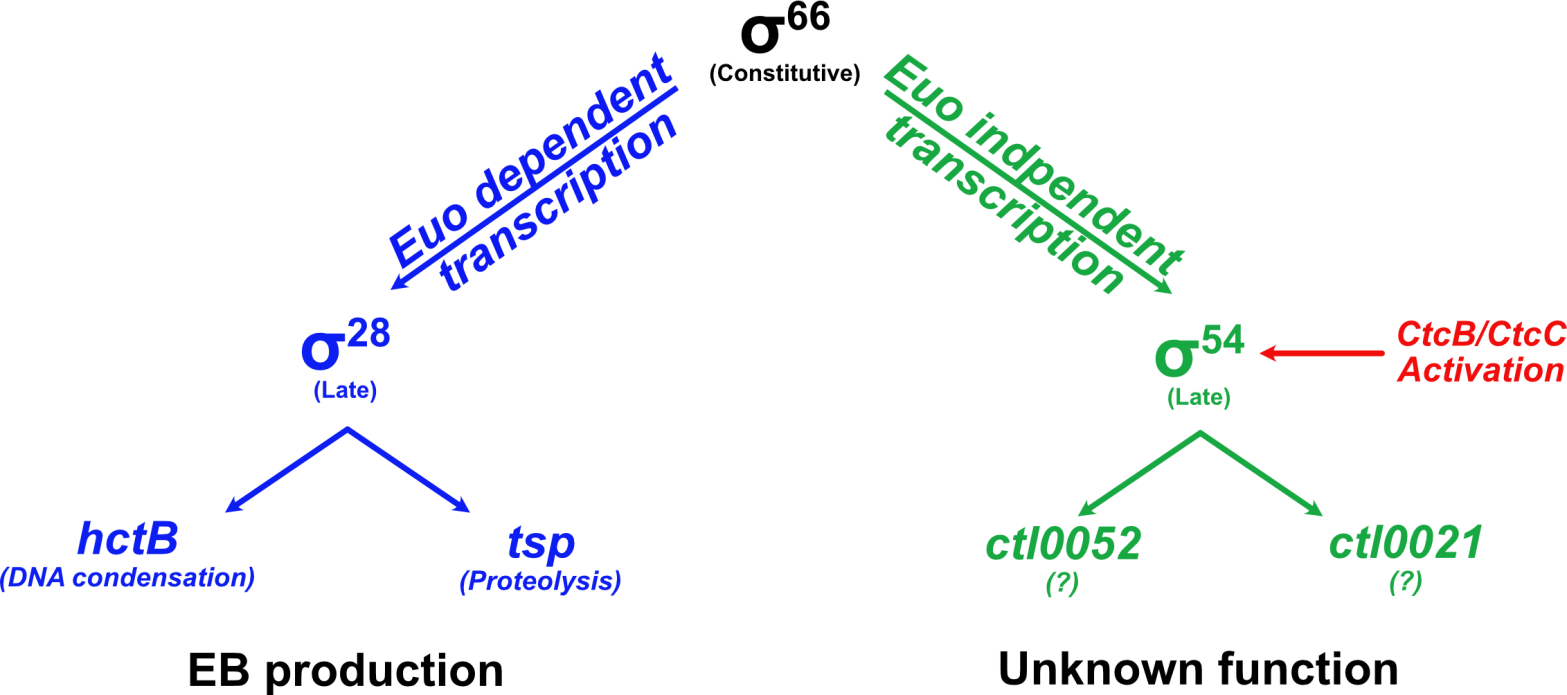
Alternative sigma factors σ^28^ and σ^54^ are under the transcriptional control of the constitutively expressed housekeeping sigma factor σ^66^, but they are expressed late. The transcriptional repressor of the late gene, Euo, represses the expression of σ^28^ before late times, acting as a temporal switch. How the late transcription of σ^54^ is regulated remains unknown. However, its activation may be controlled by an extracellular signal via the CtcB/CtcC two-component system.

One of the major insights of this study is that there are multiple mechanisms of late gene regulation in *C. trachomatis*, including dedicated mechanisms to regulate specific subsets of genes (Fig. 8). As σ^28^ and σ^54^ appear to each transcribe a limited subset of late genes, most late genes are likely transcribed by the major chlamydial RNA polymerase, σ^66^ RNA polymerase. Since this polymerase transcribes early, midcycle, and late genes, additional mechanisms are required to prevent premature expression of σ^66^-dependent late genes. Euo is a *Chlamydia*-specific transcription factor that has been shown to repress promoters of σ^66^-dependent late genes and is thus an important late regulator (11, 32). However, not all late genes are bound by Euo, so it is likely that there are additional mechanisms to control late gene expression by σ^66^ RNA polymerase (11).

While σ^28^ and σ^54^ provide mechanisms of late gene expression, they are downstream of σ^66^ in the hierarchy of late gene regulation (Fig. 8). The genes for both σ^28^ and σ^54^ are transcribed by σ^66^ RNA polymerase and therefore must be regulated by additional mechanisms to control their late expression/activity. σ^28^ expression is regulated by Euo (Fig. 3C through E) and has also been reported to be controlled by the transcriptional activator GrgA (33, 34). In contrast, we showed that the genes for σ^54^ and its activator (CtcB/CtcC) are not regulated by Euo (Fig. 6C), and thus, the mechanism that controls the late expression of σ^54^ is unknown. In addition, σ^54^ activation by CtcB/CtcC could be temporally controlled, perhaps in response to an environmental cue.

The small size of the σ^28^ and σ^54^ regulons suggests that these target genes have important roles that must be temporally regulated. The two targets of σ^28^ are Tsp and HctB, which have each been implicated in EB production. Tsp is an ortholog of tail-specific proteases in other bacteria, which are serine proteases that target and process periplasmic proteins (35). *C. trachomatis* Tsp has been shown to have both proteolytic and chaperone activity (36, 37) and was necessary for RB-to-EB conversion in *C. trachomatis* (38). However, it is not known if Tsp is essential because a *Chlamydia muridarum* temperature-sensitive *tsp* null mutant was still able to complete the developmental cycle in a cell culture infection, although with delayed EB-to-RB conversion (39). HctB is uncommon among bacterial proteins in having sequence similarity to eukaryotic histone H1 (40). This histone-like protein is only found in EBs and not RBs and binds and condenses DNA, producing a nucleoid structure and altering transcription (9, 41). The functions of the two σ^54^-regulated proteins, Ctl0021 and Ctl0052, have not been studied, but they are conserved in all *Chlamydia* (Table S1). Ctl0052 contains five tetratricopeptide repeats, which are structural motifs that mediate protein-protein interactions. This suggests that its function may involve interaction with one or more other chlamydial proteins. Ctl0021 is an uncharacterized protein conserved in all *Chlamydia* and *Chlamydia*-related bacteria with no homologs in other bacteria.

The existence of multiple mechanisms to regulate late genes in *C. trachomatis* indicates that late gene expression is a critical step in the developmental cycle. From this and previous studies, we now know that subsets of late genes are controlled by different mechanisms, including transcription factors and alternative sigma factors. This well-orchestrated control provides the means to regulate specific target genes in response to specific internal and/or environmental signals. These separate, and in some cases overlapping, control mechanisms can be viewed as locks, each with its own key, that must all be unlocked to allow the terminal differentiation of an RB into an EB. In the case of σ^28^ and σ^54^, *C. trachomatis* utilizes two dedicated forms of RNA polymerase to each transcribe just a few late genes. This exquisite level of control shows the importance of late gene regulation in controlling the production of EBs to spread intracellular infection.

## Data Availability

All sequencing data discussed in this publication have been deposited in NCBI’s Gene Expression Omnibus (GEO) (42) and are accessible through Geo Series accession numbers GSE294423 and GSE294424.

## SUPPLEMENTAL MATERIAL

Figure S1 msystems.00292-25-s0001.tiff Whole genome view of σ^28^ and σ^54^ ChIP-seq.

Table S1 msystems.00292-25-s0002.xlsx Predicted σ^54^ promoters.

ASM does not own the copyrights to Supplemental Material that may be linked to, or accessed through, an article. The authors have granted ASM a non-exclusive, world-wide license to publish the Supplemental Material files. Please contact the corresponding author directly for reuse.
