# Supplementary figures and images for: Late gene regulation by the alternative sigma factors of Chlamydia trachomatis
Source: mSystems. 2025 Jun 12;10(7):e00292-25. doi: 10.1128/msystems.00292-25 (PMC12282069; doi:10.1128/msystems.00292-25)

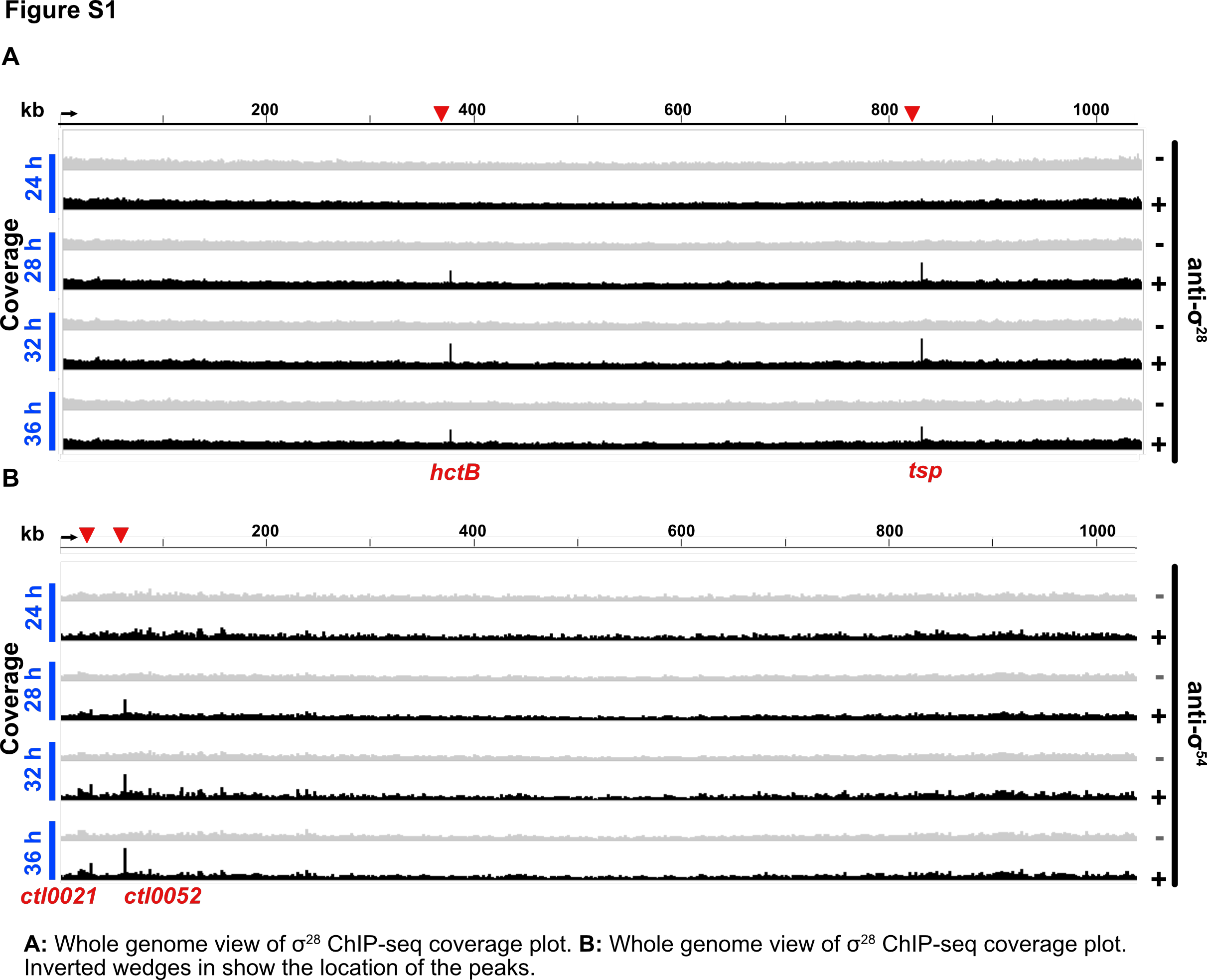

Supplement: Figure S1 — Whole genome view of σ28 and σ54 ChIP-seq. [file msystems.00292-25-s0001.tiff]
